# iTRAQ-based Comparative Serum Proteomic Analysis of Prostate Cancer Patients with or without Bone Metastasis

**DOI:** 10.7150/jca.33497

**Published:** 2019-07-10

**Authors:** Bo Yan, Binshen Chen, Shaoju Min, Yubo Gao, Yiming Zhang, Peng Xu, Chaoming Li, Jiasheng Chen, Guangheng Luo, Chunxiao Liu

**Affiliations:** 1 Department of Urology, Zhujiang Hospital, Southern Medical University, Guangzhou, Guangdong, China; 2 Department of Urology, Guizhou Provincial People's Hospital, Medical College of Guizhou University, Guiyang, Guizhou, China; 3 Department of Clinical Laboratory, The Affiliated Hospital of Guizhou Medical University, Guiyang, Guizhou, China.

**Keywords:** prostate cancer, bone metastasis, quantitative proteomics, iTRAQ labeling

## Abstract

**Background**: Once prostate cancer developed bone metastasis, the quality of life and prognosis of patients are seriously affected as no effective treatment is currently available. It is necessary to explore the mechanism of bone metastasis and new therapeutic targets.

**Purpose**: To find out the differentially expressed serum proteins in prostate cancer patients with bone metastasis and analyze the expression of key proteins in prostate cancer tissues, serum and prostate cancer cell lines. So as to provide a basis for revealing the mechanism of bone metastasis and designing new therapeutic targets.

**Methods**: iTRAQ-based proteomics method was used to compare serum differential proteins in prostate cancer patients with and without bone metastasis. Three key proteins (CD59, haptoglobin and tetranectin) which had significant fold changes were selected to validate the results of mass spectrometry. Immunohistochemistry and ELISA were applied to tissues and serum samples from prostate cancer patients, respectively, for validation. Finally, western blot, flow cytometry, and immunocytochemistry were used to analyze the expression of the three differentially expressed proteins in the prostate cancer cell lines PC3, LNCap, and Du145.

**Results**: Thirty-two differentially expressed proteins related to bone metastasis of prostate cancer were identified, of which 11 were up-regulated and 21 were down-regulated. CD59 and haptoglobin were up-regulated in prostate cancer with bone metastasis while tetranectin was down-regulated. Tetranectin showed differential expression in epithelial and stromal cells of prostate cancer and hyperplasia tissues.The expression of CD59 was highest in PC3 and lowest in LNCap, while the expression of haptoglobin and tetranectin was the highest in DU145 and lowest in PC3.

**Conclusion**: Mass spectrometry analysis showed that there were more differentially expressed proteins in the serum of patients with bone metastasis than those without metastasis. It has been verified that CD59, haptoglobin and tetranectin are prostate cancer bone metastasis related proteins.

## 1. Introduction

Prostate cancer is one of the most common malignancies in Europe and the United States, with a mortality rate that is second only to lung cancer 1. Worldwide, the incidence of prostate cancer ranks second among male malignancies 2. Prostate cancer has the highest likelihood of metastasizing to the bone. About 65-80% of prostate cancer patients have bone metastasis in advanced stage 3, 4. However, the specific mechanism leading to bone metastasis is still unclear. Improvements in treatment strategies in recent years have increased the 5-year survival rate of patients with localized prostate cancer to over 90%; however, once the tumor cells spread to specific secondary organs such as the bone, the 5-year survival rate significantly decreases to only 28% 5. Along with poor prognosis, the quality of life of prostate cancer patients with bone metastasis is greatly affected because bone metastasis can lead to bone pain, hypercalcemia, pathological fractures and/or nerve compression 5. At present, there is no ideal treatment or cure for bone metastasis of prostate cancer 6. Thus, exploring the molecular mechanism of bone metastasis of prostate cancer and identifying new therapeutic targets has been the focus of many recent studies.

Bone metastasis of prostate cancer is a complex biological process affected by various factors. Bone is the third most common site of metastasis, second only to lung and liver, suggesting that bone/marrow has a favorable microenvironment for tumor growth 7. Prostate cancer is prone to bone metastasis for two reasons. Firstly, the intrinsic biological properties of bone/marrow microenvironment provide natural favorable conditions for prostate cancer to colonize and proliferate in bone. Second, prostate cancer cells can secrete a variety of cytokines to regulate bone/marrow microenvironment and make it more suitable for their own growth 8, 9. Under normal physiological conditions, bone remodeling releases a large number of growth factors, cytokines, and cell adhesion factors into the bone microenvironment, which can attract prostate cancer cells through the process of chemotaxis 10, 11. The most important of these factors is stromal-derived factor-1 (SDF1), also known as chemokine ligand-12, expressed by bone marrow endothelial cells. SDF1 binds to chemokine receptor-4, a highly expressed receptor on the surface of cancer cells, causing the tumor cells to migrate and adhere to bone marrow extracellular matrix 12. When prostate cancer cells migrate to bone/marrow microenvironment, they interact with osteoblasts, osteoclasts, bone marrow mesenchymal stem cells and hematopoietic stem cells through various signaling pathways to form a complex regulatory network. Currently, there are some reported signaling pathways that may be associated with bone metastasis of prostate cancer, such as mesenchymal to epithelial transition factor pathway, vascular endothelial growth factor pathway, β2-adrenergic receptor pathway, androgen receptor pathway, and RANK/RANKL/OPG signaling pathway 13-17. A clinical study has shown that denosumab, an IgG2 antibody that binds human RANKL with high affinity, can prolong the occurrence of bone metastasis for the first time in prostate cancer (CRPC) patients 18. This study is the first successful evidence that blocking key signaling pathways in bone microenvironment can delay bone metastasis by making the microenvironment unsuitable for tumor cell colonization. Although some encouraging results have been achieved, the molecular mechanism of bone metastasis in prostate cancer is not fully elucidated, and further research is needed.

In this study, isobaric tags for relative and absolute quantitation (iTRAQ)-based quantitative proteomic technology was used to analyze the differentially expressed proteins (DEPs) in the serum of prostate cancer patients with bone metastasis and non-metastasis. Three such DEPs with large fold changes were selected for further analysis and validation using immunohistochemistry and ELISA. We also analyzed the expression of the three DEPs in three commonly used prostate cancer cell lines. Our results provide new clues for further study of the role of these DEPs in the occurrence and development of bone metastasis of prostate cancer and for exploring possible therapeutic targets.

## 2. Materials and Methods

### 2.1 Materials

#### 2.1.1 Serum samples

Clinical samples and data collected comply with the regulations of China Medical Ethics Committee. All sample contributors had signed informed consent. The serum samples of 30 prostate cancer patients without metastasis and 30 prostate cancer patients with bone metastasis were collected from the urology department of Zhujiang Hospital of Southern Medical University. All of them were confirmed by prostate biopsy. No castration therapy was performed before blood collection in all patients. Bone metastasis were confirmed by single photon emission computed tomography and magnetic resonance imaging. The age of the patients ranged from 57-73 years, with the average age being 65. Five milliliters whole blood samples were collected without anticoagulant. Samples were stored in refrigerator at 4°C for 2 h and then centrifuged at 4°C for 10 min at 3000 rpm. The supernatant was collected and stored at -80°C.

### 2.2 Methods

#### 2.2.1 Experimental design

Prostate cancer with bone metastasis group and without metastasis group each consisted of 30 cases, giving 30 serum samples. Each group was divided into two subgroups, each subgroup consisting of 15 cases. The 15 serum samples of each subgroup were mixed together with equal volume to extract and purify the protein. The samples from cases with bone metastasis were labeled with iTRAQ114 and 121 and those without metastasis were labeled with iTRAQ116 and 118 respectively (iTRAQ reagents were procured from AB Sciex, California, USA). Experiment was performed in two replicates, i.e. 4 repetitions in each group. Repeated manipulation begins at the stage of enzymatic hydrolysis and labeling of proteins.

#### 2.2.2 Protein extraction and purification

The serum samples were thawed and purified with ProteoPrep blue albumin & IgG depletion Kit (Sigma, Germany). A 4-fold volume of acetone was added to the purified filtrate and then left for precipitation overnight at -20°C. The precipitated protein was centrifuged at 12000 rpm, for 20 minutes, at 4°C. The supernatant was removed and the pellet was dried. L3 lysis buffer (200 μL) was added to the dried protein lumps, and then dissolved by ultrasonic assistance, and centrifuged at 12000 rpm, for 20 min at 4°C. The supernatant was transferred to new microcentrifuge tubes and protein quantification was performed using BCA Protein Assay Kit (Thermo Fisher, USA).

#### 2.2.3 Protein digestion and labeling with iTRAQ reagents

To 200 µg protein solution, 4 µL TCEP Reducing Reagent was added, and the reaction was incubated at 60°C for 1 h. Post-incubation, 2 µL MMTS Cysteine-Blocking Reagent was added, and the reaction was placed in 25°C for 30 min. The reduced alkylated protein solution was added into a 10K ultrafiltration tube (Merck Millipore, Germany) and centrifuged at 4°C for 20 min, after which the solution in the collection tube was discarded. Then, 100 µL 8 M urea was added and the bottom solution in the collection tube was discarded after centrifugation at 12 000 rpm for 20 min at 4°C; this step was repeated twice. Next, 100 µL of 0.25 M tetraethylammonium tetrahydroborate (TEAB) was added and samples were centrifuged at 12000 rpm for 20 min at 4°C, after which the solution in the collection tube was discarded; this step was repeated three times. The collection tube was then replaced with a new one, and 50 µL 0.5 M TEAB and trypsin (trypsin to protein mass ratio 1:50) was added into the ultrafiltration tube for overnight reaction at 37°C. Trypsin was added (trypsin to protein mass ratio 1:100) and reaction was kept at 37°C for 4 h and then centrifuged at 12000 rpm for 20 min at 4°C. After adding 50 µL 0.5 M TEAB into the ultrafiltration tube and centrifuging at 4°C 12000 rpm for 20 min, 100 µL samples were obtained at the bottom of the collection tube.

Isopropanol (150 µL) was added to the iTRAQ reagents in a microcentrifuge tube, vortexed and centrifuged. Samples (50 µL; 100 µg enzymatic hydrolysate) were transferred to a new microcentrifuge tube and the iTRAQ reagent was added to the sample. After vortexing and a short spin, the samples were left at 25°C for 2 h, after which the reaction was terminated by adding 100 µL water. The labeled samples were then dried by vacuum freeze-centrifugation.

#### 2.2.4 High-pH reversed-phase chromatography

Before high-performance liquid chromatography (Shimadzu HPLC, Moldel: LC-20AD), Phenomenex column (Gemini-NX 3u C18 110A, 150 × 2.00mm, USA) was equilibrated with 95% buffer A (H_2_O, NH_3_ in H_2_O, pH = 10) and 5% buffer B (80% acrylonitrile [ACN],NH_3_ in H_2_O, pH = 10) for 30 min. The dried iTRAQ-labeled peptide samples were then dissolved in 200 µL buffer A solution and loaded onto the Phenomenex column. Peptides were separated by a linear gradient formed from buffer A and buffer B, from 5% to 95% of buffer B at a flow rate of 0.2 mL/min. The whole elution process was monitored using 214 nm ultraviolet light and 15 fractions were collected from the linear gradient according to the peak shape. These fractions were analyzed by LC-MS/MS after vacuum drying.

#### 2.2.5 Liquid chromatography coupled with tandem mass spectrometry

Peptide fractions collected through HPLC were dissolved in formic acid (FA)-acrylonitrile (ACN) solution (2% ACN, 0.1% FA), thoroughly vortexed, and centrifuged at 13,500 rpm for 20 min at 4°C. The supernatant was transferred to the loading tube, and 3 µg sample from every fraction was taken for further separation by two-dimensional liquid chromategraphy using a Dionex column( Acclaim PepMap RSLC C18, 75μm i.d. x 150mm ). Peptides were separated by a linear gradient formed from mobile phase A (0.1% FA, 5% ACN) and mobile phase B (0.1% FA, 95% ACN), from 5% to 90% of mobile phase B for 65 min, at a flow rate of 300 nL/min. The separated peptides directly entered the mass spectrometer (Thermo Scientific, Modle: Q Exactive) for detection. MS spectra were acquired across the range of 350-1800 m/z in high resolution mode (70,000), using 100 ms accumulation time per spectrum. A maximum of 20 precursors per cycle were chosen for fragmentation from each MS spectrum with 100 ms minimum accumulation time for each precursor and dynamic exclusion for 20 s. Tandem mass spectra were recorded in high sensitivity mode (resolution : 17500), with turned on rolling collision and iTRAQ reagent collision energy adjustment. The protein quantification was based on unique peptides.

#### 2.2.6 Protein retrieval and differential protein screening

Mass spectrometry data in RAW format were acquired by mass spectrometer. The RAW file was converted into mascot generic format (MGF) file by Thermo Proteome Discoverer. The MGF file and protein retrieval library were inputted into the ProteinPilot Software^TM^ 4.5 (UniProt_Sp_Human_2017.11.01.fasta, 20226 proteins reviewed) for protein retrieval. A strict cutoff for protein identification was applied with an unused ProtScore ≥ 1.3, which corresponded to a confidence limit of 95%, to minimize false positive results. At least two peptides with 95% confidence and coefficient variation (CV) < 0.5 were considered for protein quantification. False discovery rate (FDR) analysis was performed using the integrated tools in ProteinPilot (FDR < 0.01). For data analysis, protein quantification data with a fold change of > 1.3 and < 0.77 and *p*-value < 0.05 was selected as the significantly differently expressed protein (DEP). The interaction network of DEPs was analyzed by String online tool.

#### 2.2.7 Immunohistochemical staining and semi quantitative analysis

Pathological sections including 5 cases of bone metastasis tissue derived from prostate cancer, 30 cases of prostate cancer with bone metastasis, 30 cases of non-metastatic prostate cancer, and 30 cases of benign prostatic hyperplasia were collected. Three sections were prepared of every case and stained with antibodies for CD59, haptoglobin and tetranectin, respectively. Unstained sections were de-paraffinized and rehydrated using standard methods. Endogenous peroxidase activity was quenched by slide immersion in 3% hydrogen peroxide solution for 10 min followed by a PBS rinse. For antigen retrieval, slides were incubated in citric acid antigen repair solution in a steamer for 8 min at 100°C, followed by cooling down to 25°C naturally and a PBS rinse. Normal goat serum (10%) was placed on sections for 30 min for antigen blocking. Slides were incubated in primary antibody diluted in 10% goat serum overnight. The following primary antibodies and concentrations were used: anti-CD59 [Abcam, ab9182; mouse monoclonal (1:100)], anti-haptoglobin [Abcam, ab13429; mouse monoclonal (1:100)]; anti-tetranectin [Abcam, ab51883; mouse monoclonal (1:150)]. Slides were rinsed in PBS followed by incubation in secondary antibody [Abcam, rabbit anti-mouse IgG, ab6728 (1:200)] for 30 min. Diaminobenzidine (DAB) detection, hematoxylin counterstaining, dehydration and sealing were performed according to conventional methods.

Image-pro Plus 6.0 software was used to calculate the optical density of the area of interest in each slice, and the average optical density of each group was compared. Haptoglobin and CD59 were only expressed in glandular epithelial cells; thus, glandular epithelium was selected as the area of interest. Tetranectin was expressed in both glandular epithelium and stroma; thus, the glandular epithelium and stroma area were both analyzed for tetranectin.

#### 2.2.8 ELISA

Serum samples were collected from 50 patients with prostate cancer and bone metastasis, 50 patients with prostate cancer without metastasis and 50 patients with benign prostatic hyperplasia. The Human CD 59 ELISA Kit (Ray Biotech , Norcross, USA), Human Haptoglobin Quantikine ELISA Kit (R&D, Minneapolis, USA), and Custom Human CLEC3B ELISA Kit (Ray Biotech, Norcross, USA) were used to detect the levels of CD59, haptoglobin and tetranectin, respectively. All operations were carried out in strict accordance with the manufacturer's operation manual.

#### 2.2.9 Cell culture

PC3, LNCap, and Du145 human prostate cancer cell lines used in this study were purchased from American Type Culture Collection. LNCaP cell line is derived from lymph node metastasis of prostate cancer patients and has low metastasis potential, representing the growth characteristics of early androgen-dependent prostate cancer. PC3 cell line is derived from bone metastasis of prostate cancer and has moderate metastasis potential. DU145 cell line is derived from brain metastasis tissue of prostate cancer patients and has strong metastasis potential. Cells were cultured in RPMI-1640 medium (Sigma-Aldrich, St.Louis, USA), supplemented with 10% FBS (Gibco, California, USA), 1% antibiotic-antimycotic (Gibco, California, USA), and 1% glutaMAX (Gibco, California, USA) at 37°C and 5% CO_2_. The cell lines were authenticated using short-tandem repeat profiling provided by the vendor and routinely monitored for mycoplasma contamination^19^. Cells were routinely passed by treatment with trypsin (0.05%)/EDTA.

#### 2.2.10 Flow cytometry

Cells were harvested by incubation with TrypLE for 3 min at 37°C. Each group of cells was divided into negative control and test group with each tube containing a total of 100 μL. The cell concentration was adjusted to 1×10^6^/100 μL. Primary antibody (1 μL) was added to the test tube and incubated at 4°C for 30 min. All tubes, including blank control tube, were washed with 500 μL PBS and then treated with anti-mouse IgG (whole molecule)-FITC antibody produced in goat (Sigma-Aldrich, St.Louis, USA) and incubated at 4°C for 30 min. The samples were then washed with 500 μL PBS and re-suspended in flow cytometry staining buffer (eBioscience, Carlsbad, USA). All samples were analyzed on a FACSCalibur flow cytometer (Becton-Dickinson, USA).

#### 2.2.11 Western blot

The cell lysates were prepared by lysis buffer consisting of 50 mM Tris-HCl buffer (pH 7.4), supplemented with 1% Triton X-100, 150 mM NaCl, 1 mM EDTA, 0.5% sodium deoxycholate, 0.1% SDS, 1 mM PMSF, and HALT™ protease inhibitor cocktail at 1× concentration. Protein concentrations were determined using BCA Protein Assay Kit (Thermo Fisher, Rockford, USA). 40μg of lysate proteins were denatured with loading buffer by boiling for 5 min. The denatured lysates were then resolved by SDS-polyacrylamide gel electrophoresis. The separating gel concentration used for haptoglobin, tetracycline and CD59 were 8%, 12% and 15% respectively. The stacking gel concentration used for all three proteins was 5%.The resolved proteins were transferred to PVDF membranes (Merck Millipore, Temecula, USA). The membranes were blocked with 5% skimmed milk solution for 1 h at 25°C and washed three times in TBS-Tween. The blocked membranes were probed overnight at 4°C with the primary antibody solution (1:1000). The primary antibody used is the same as the immunohistochemical part. After washing three times in TBS-Tween, the blots were probed with the corresponding secondary antibody (1:3000) conjugated with horse radish peroxidase for 1 h at 4°C. After the final washes, the signal was developed with the Immobilon Western Chemilum HRP substrate (Merck Millipore, Temecula, USA), as recommended by the manufacturer, and the bands were captured on to Carestream autoradiography film (Carestream Health, Inc. Canada).

#### 2.2.12 Immunofluorescence cytochemistry

Coverslips were coated with 0.5% gelatin (Sigma-Aldrich, St. Louis, USA) and three different cell lines of prostate cancer were seeded onto different coverslips and cultured in RPMI-1640 medium supplemented with 10% FBS. When cells grew to 70% confluence on the coverslips, they were fixed with 4% paraformaldehyde for 30 min and incubated with 0.2% Triton X-100 for 5 min at 25°C. The cells were then treated with 10% normal goat serum (Abcam, ab7481), and incubated with primary mouse anti-human antibodies against CD59, Haptoglobin, and Tetranectin (all from Abcam,the same catalog number as immunohistochemistry) at a dilution of 1:100 at 4°C overnight, followed by Alexa Fluor® 488-conjugated goat anti-mouse antibody at a dilution of 1:200 (Abcam, ab150113) at 25°C for 1 h in the dark. After staining with DAPI (CST, Danvers, USA) for 5 min to visualize the nuclei, cells were imaged by fluorescence microscopy (Olympus, Japan).

#### 2.2.13 Statistical analysis

The optical density of immunohistochemical sections and the concentration of each index measured by ELISA in each group are expressed as mean ± standard error. Statistical comparisons between groups were carried out using one-way ANOVA. Results were considered statistically significant at the *p*-value < 0.05.

## 3. Results

### 3.1 Differentially expressed proteins

Based on the screening conditions mentioned above (Section 2.2.6), 32 differentially expressed proteins (DEPs) were identified, of which 11 were up-regulated and 21 were down-regulated in samples obtained from the prostate cancer with bone metastasis group. All up and down-regulated DEPs are shown in Table 1.

### 3.2 Interaction network analysis of differential proteins

**Fig 1:**
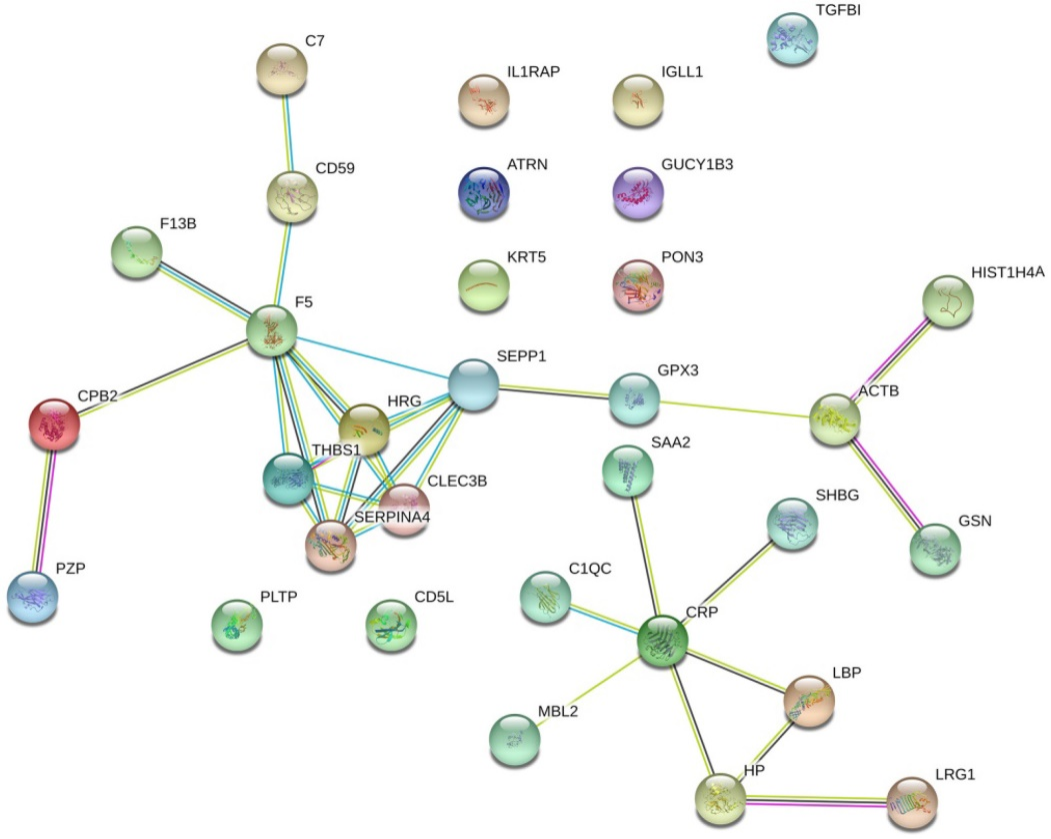
The protein interaction network of 32 bone metastasis-related proteins of prostate cancer analyzed by STRING online tool. Only the F5, SEPP1, THBS1, HRG, SERPINA4, and CLEC3B were shown to interact with each other in the whole network. No other DEPs formed an interaction network.

The protein interaction network of the 32 prostate cancer bone metastasis-related proteins was analyzed by the STRING online tool. The results showed that F5, SEPP1, THBS1, HRG, SERPINA4, and CLEC3B interacted with each other in the whole network, but no other DEPs formed an interaction network (Fig. 1).

### 3.3 Immunohistochemistry

**Fig 2:**
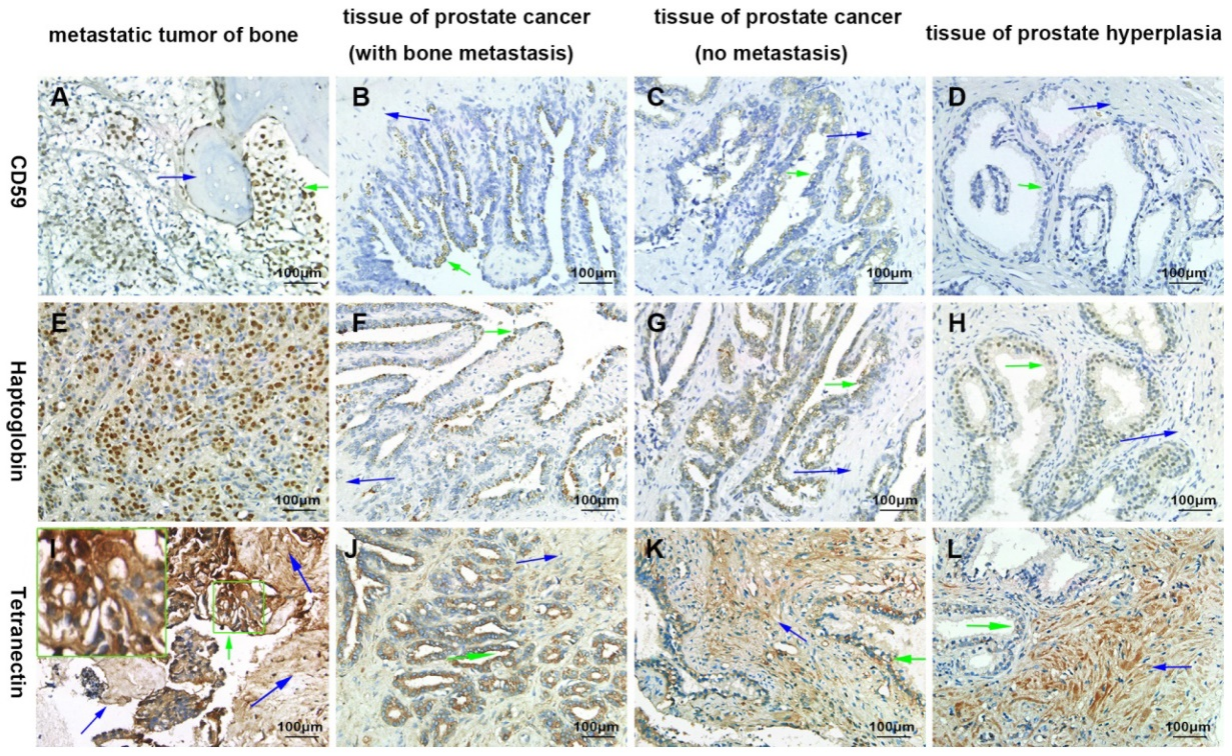
Confirmation of the differential expression of CD59, haptoglobin, and tetranectin by immunohistochemical analysis. The green arrows indicate the prostate cancer or proliferating epithelial cells. The blue arrows indicate bone tissues in A) and I), and indicates the prostatic stroma in other graphics. All pictures were 400× magnified.

**Fig 3:**
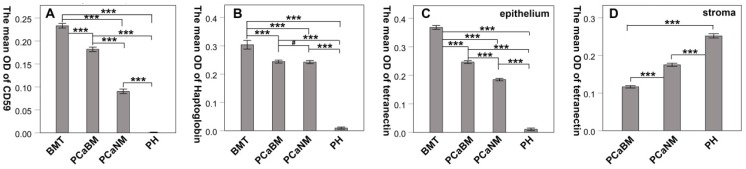
The mean optical density of CD59, haptoglobin and tetranectin in prostate cancer and hyperplasia tissues. BMT: bone metastatic tumor; PCaBM: prostate cancer with bone metastasis; PCaNM: prostate cancer with non-metastasis; PH: prostate hyperplasia. *P < 0.05, ***P < 0.001, ^#^*P* > 0.05.

By immunohistochemical staining, we found that CD59 was expressed in cell membrane and cytoplasm of prostate cancer epithelial cells, and positive staining particles were found in the cytoplasm. CD59 was most strongly expressed in bone metastatic tumor of prostate cancer, but rarely expressed in glandular epithelial cells of prostate hyperplasia, which was nearly negative for CD59 (Fig. 2). The mean optical density values of bone metastatic tumor, prostate cancer tissue with bone metastasis, prostate cancer tissue with no metastasis, and benign prostatic hyperplasia tissue were 0.233 ± 0.005, 0.182 ± 0.005, 0.090 ± 0.005, and 0.001 ± 0.000, respectively (Fig. 3). The difference was statistically significant between each group (*p* < 0.001).

Haptoglobin was also expressed in prostate cancer epithelial cells, with positive expression in nucleus and cytoplasm. There were many positive staining granules around the cells. The intensity of haptoglobin expression in bone metastatic tumor was significantly higher than that in prostate cancer tissue with or without bone metastasis. The expression of haptoglobin in prostate hyperplasia was the weakest (Fig. 2). The mean optical density values of bone metastatic tumor, prostate cancer tissue with bone metastasis, prostate cancer tissue with no metastasis, and benign prostatic hyperplasia tissue were 0.304 ± 0.015, 0.244 ± 0.005, 0.242 ± 0.006, and 0.009 ± 0.004, respectively (Fig. 3). There was no significant statistical difference between prostate cancer tissue with bone metastasis and non-metastasis group (*p* =0.767), while significant differences were found between the other groups (*p* < 0.001).

Tetranectin was expressed in both cytoplasm of epithelial cells and mesenchyme. In epithelial cells, its expression in bone metastatic tumor, prostate cancer tissue with bone metastasis, prostate cancer tissue without metastasis, and benign prostatic hyperplasia tissue decreased in that order (Fig. 2). The mean optical density values of each group were 0.368 ± 0.007, 0.246 ± 0.005, 0.185 ± 0.004, and 0.011 ± 0.004, respectively (Fig. 3) and significant statistical differences were observed between the groups (*p* < 0.001). Since the bone metastasis tumor tissues were all dense prostate cancer epithelial cells, it was impossible to analyze the optical density of the mesenchyme, so only three groups of data were analyzed. Thus, the mean optical density in prostate cancer tissue with bone metastasis, prostate cancer tissue with no metastasis, and benign prostatic hyperplasia tissue was 0.112 ± 0.003, 0.175 ± 0.004, and 0.252 ± 0.006, respectively (Fig. 3). The intensity of expression in the mesenchyme increased in the above-mentioned order. Significant statistical differences were observed between the groups (*p* < 0.001).

### 3.4 ELISA

**Fig 4:**
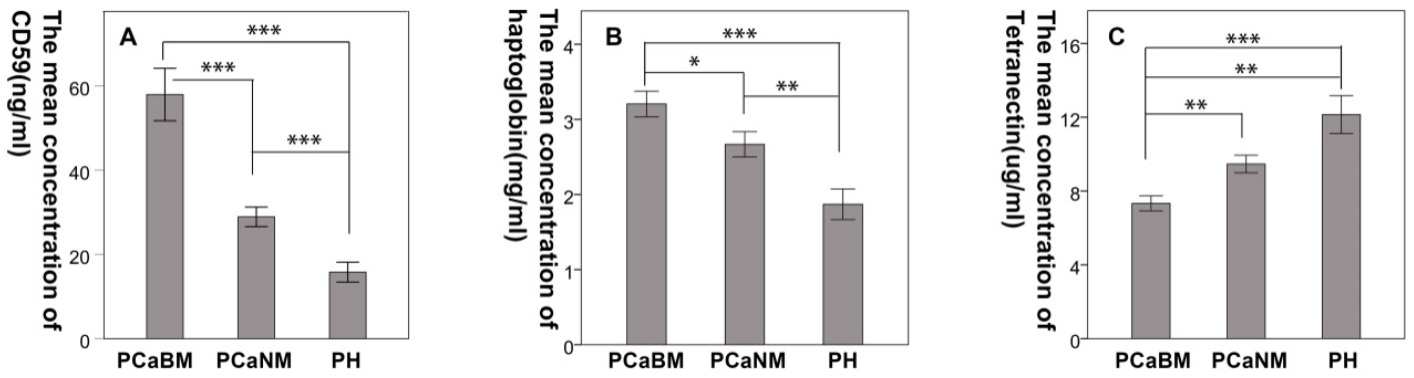
Confirmation of the differential expression of CD59, haptoglobin, and tetranectin in serum by ELISA. PCaBM: prostate cancer with bone metastasis; PCaNM: prostate cancer with non-metastasis; PH: prostate hyperplasia. *P < 0.05, **P < 0.01, ***P < 0.001.

The mean CD59 concentrations (in ng/mL) in serum of prostate cancer patients with bone metastasis, prostate cancer without metastasis and benign prostatic hyperplasia were 57.94 (± 17.05), 28.92 (± 6.35), and 15.79 (± 5.29), respectively. The mean haptoglobin concentrations (in mg/mL) were 3.21 (± 0.93), 2.67 (± 0.92), and 1.87 (± 0.91), respectively. The mean concentrations of tetranectin (in μg/mL) were 7.33 (± 2.23), 9.47 (± 2.62), and 12.15 (± 4.59), respectively. The differences were statistically significant among the three groups for each protein (*p* < 0.05) (Fig. 4).

### 3.5 Immunofluorescence and flow cytometry

**Fig 5:**
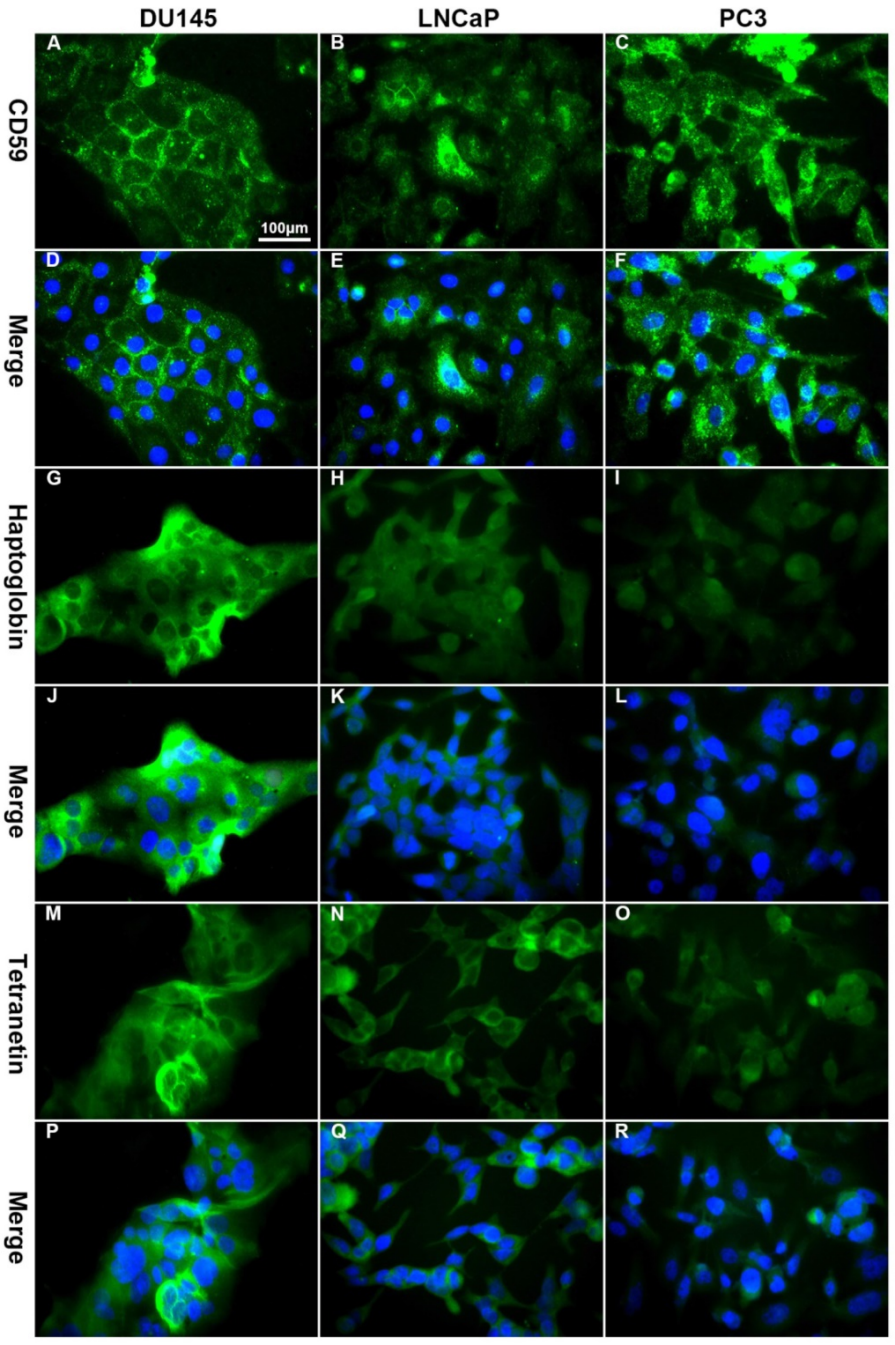
The differential expression of CD59, haptoglobin, and tetranectin in different cell lines analyzed by immunofluorescence. The expression of CD59 was highest in PC3 and lowest in LNCap, while the expression of haptoglobin and tetranectin was the highest in DU145 and lowest in PC3. All pictures were 400× magnified. The scale bar in A is applicable to all pictures.

**Fig 6:**
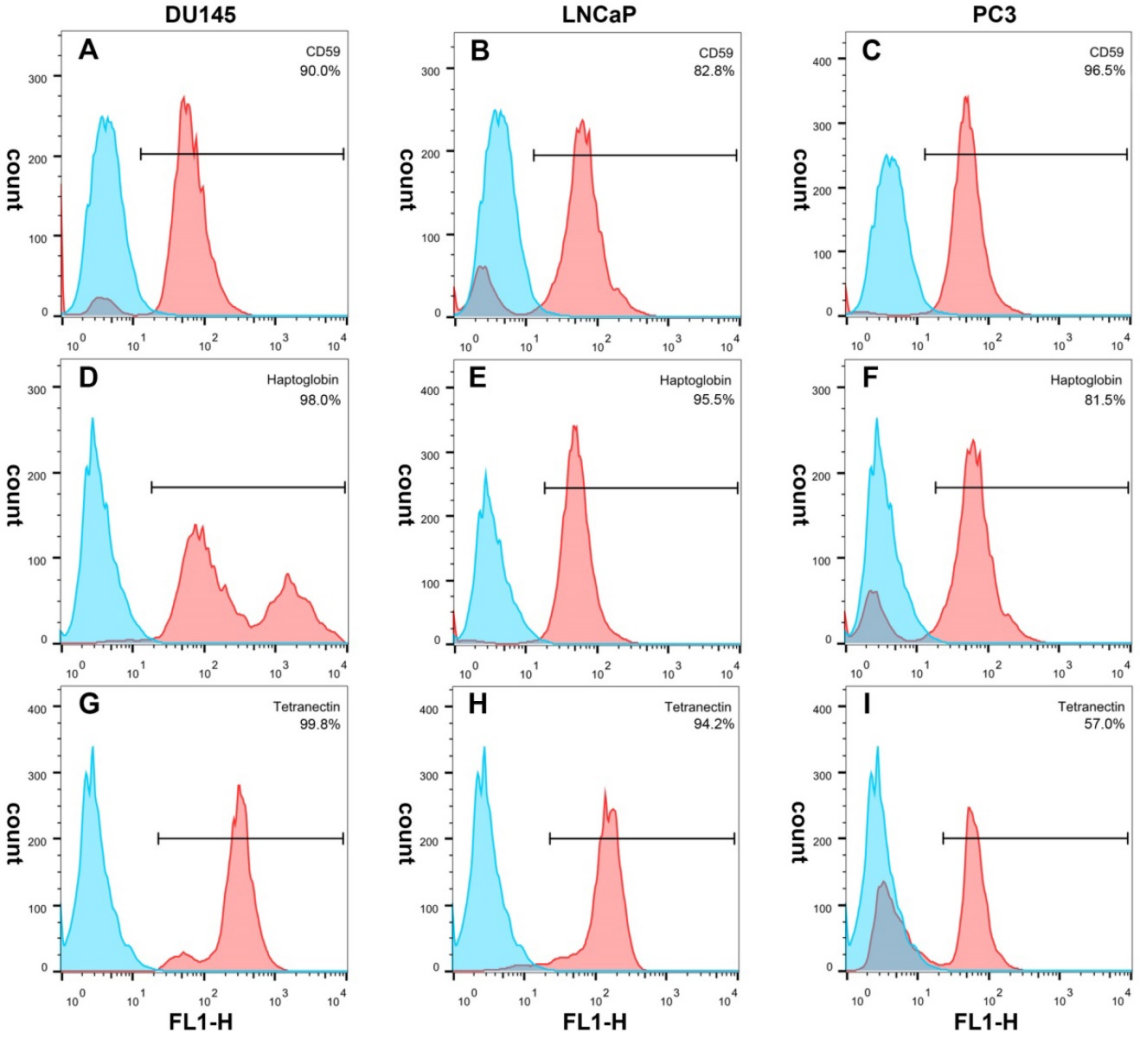
The positive expression ratio of CD59, haptoglobin, and tetranectin in three different cell lines analyzed by flow cytometry. The right upper corner of each picture shows the detected protein and the ratio of positive stained cells.

Immunofluorescence showed that CD59 was mainly expressed on the cell membrane, although granular positively stained particles were also found in the cytoplasm. CD59 expression was highest in the PC3 cell line and lowest in the LNCaP. Haptoglobin and tetranectin were mainly expressed in the cytoplasm and their expression was highest in DU145 and lowest in PC3 cell line (Fig. 5). Flow cytometry indicated that the positive rate of CD59 was highest in PC3 and lowest in LNCaP, while haptoglobin and tetranectin were highest in DU145 and lowest in PC3 (Fig. 6).

### 3.6 Western blot

**Fig 7:**
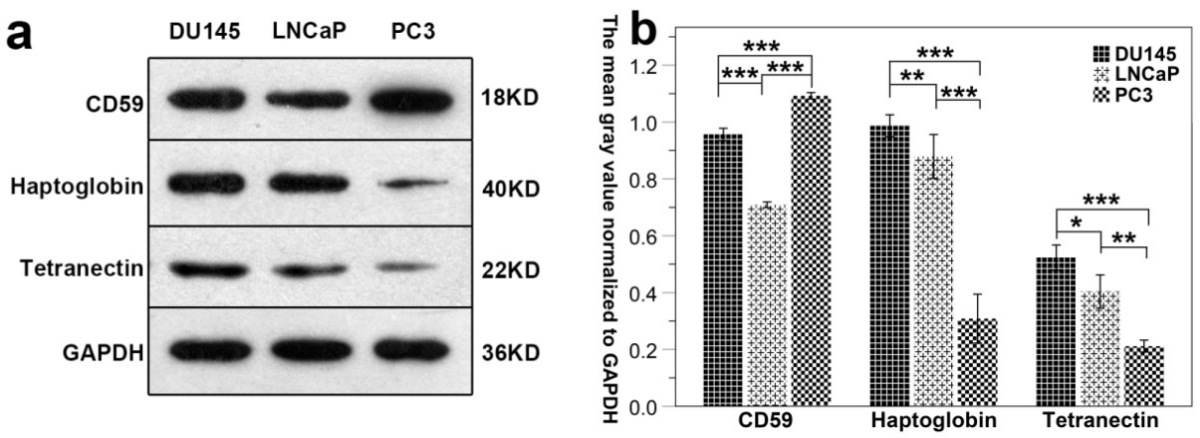
The differential expression of CD59, haptoglobin, and tetranectin in different cell lines. Fig.7a. The expression of three proteins in three different cell lines analyzed by Western Blot. Fig.7b. The bar graphs represent the data after normalization to GAPDH. *P < 0.05, **P < 0.01, ***P < 0.001.

Results show that the expression of CD59 was highest in PC3 cell line and was lowest in LNCaP. The expression of haptoglobin and tetranectin was highest in DU145 cell line and was lowest in PC3. The expression of the three proteins in the three different cell lines is shown in Fig. 7a. The mean gray density of bands from three experimental replicates is shown in Fig. 7b.

## 4. Discussion

The occurrence of bone metastasis of prostate cancer is a complex process. Due to the lack of an ideal animal model of bone metastasis of prostate cancer, the research progress on understanding the metastasis mechanism has been slow. Although several studies have reported multiple methods of generating nude mice model of bone metastasis of prostate cancer, some of these methods cannot completely simulate the pathophysiological process of bone metastasis in the human body, and are therefore not ideal. Some methods can better simulate the pathophysiological process of bone metastasis, but these are difficult and the success rate has been very low. In this study, we screened for and identified 32 bone metastasis-related serum proteins from patients with prostate cancer. Of these, CD59, haptoglobin, and tetranectin showed a significantly large fold change in patients with bone metastasis, which we further verified through immunohistochemistry and ELISA.

CD59 is a glycosylphosphatidylinositol-anchored membrane protein that acts as an inhibitor of the membrane attack complex formation to regulate complement activation 20. CD59 is widely expressed in various organs and tissues, including skin, liver, kidney, pancreas, lung, salivary gland, heart, nervous system, placenta, vascular endothelial cells, various blood cells (red blood cells, lymphocytes, neutrophils and platelets) and sperm 21. CD59 is the only inhibitory protein among the membrane-bound complement regulatory proteins that acts on the activation end-stage of the complement system. It can inhibit the formation of the complement membrane attack complexes, thus effectively protecting the host cells from MAC-induced cell lysis; thus, it plays an important role in the mechanism of tumor immune escape. In our study, mass spectrometry analysis showed that the expression of CD59 in serum of prostate cancer patients with bone metastasis was significantly higher than that in non-metastasis patients, and the results were validated by serum ELISA analysis of a larger sample size. The immunohistochemical staining showed that CD59 was expressed in bone metastasis tissues and prostate cancer tissues, and almost negative in prostate hyperplasia tissues. The positive expression sites were on epithelial cells, while the mesenchyme was negative for CD59 expression.The expression intensity of CD59 was found progressively lower going from the bone metastatic tumor group to the prostate cancer with bone metastasis, prostate cancer without metastasis and prostate hyperplasia groups Therefore, we speculate that the increased expression of CD59 may prevent prostate cancer cells from being attacked by the immune system and facilitate their colonization in bone/marrow tissues, thereby leading to bone metastasis. A large number of studies confirmed that downregulation of CD59 expression or use of CD59 inhibitors can improve the therapeutic effect of antibodies on tumors 22. Overexpression of CD59 can increase the expression of anti-apoptotic protein Bcl-2 and promote tumor cell growth and proliferation 23. It is reported that CD59 inhibitors (such as MB59 monoclonal antibody or rILYd4 protein) can significantly inhibit the function of CD59, and thus improve the therapeutic effect of rituximab on B-cell lymphoma and chronic lymphoblastic leukemia 24, 25. Thus, CD59 is a promising new target for cancer immunotherapy 20.

In this study, we analyzed the expression of CD59 in prostate cancer cell lines DU145, LNCap and PC3. The results showed that CD59 had the highest expression in PC3. Because PC3 cell line is from bone metastasis of a prostate cancer patient, this indicates that CD59 has a high correlation with bone metastasis of prostate cancer. Recent studies suggested that the cytokines in the tumor microenvironment, such as TNF-α, IL-1β, IL-6 and IFN-γ, could influence the expression of CD59 on tumor cells 26, 27. However, it is unclear how these cytokines regulate the expression of CD59 in tumor cells. Moreover, the functions of soluble CD59 and glycosylated CD59 needs further investigation.

Haptoglobin is a plasma high-abundance acidic glycoprotein that is mainly synthesized by the liver; other tissues such as skin, lung and kidney have a low level of expression. The synthesized haptoglobin can be secreted into the blood circulation system. The normal haptoglobin plasma concentration is in the range of 0.5-3 g/L 28. The haptoglobin concentration may increase substantially during acute inflammation in response to the acute phase mediator interleukin-6 (IL-6) and other cytokines 29. The main function of haptoglobin is to bind free hemoglobin (Hb) to form a stable haptoglobin-Hb complex. Through the mediation of scavenger receptor CD163, haptoglobin can be quickly endocytosed into the monocyte-macrophage system to prevent the loss of iron and hemoglobin from the kidney 30, and protect DNA and tissues from oxidative damage of heme 31. It also functions in regulating T cell immune response, cell proliferation, angiogenesis and arterial reconstruction. Haptoglobin can promote malignant transformation of epithelial cells and inhibit tumor immunity 32. It has been reported that haptoglobin is associated with the occurrence and development of a variety of tumors, including lung cancer 33, pancreatic cancer 34, and liver cancer 35. In this study, we confirmed that the expression of haptoglobin was significantly higher in prostate cancer (with or without bone metastasis) and bone metastasis tissues than in prostate hyperplasia. It is concluded that the expression of haptoglobin is correlated with the occurrence of prostate cancer and bone metastasis, which is consistent with previous reports. Dempsey et al. 36 reported that haptoglobin interacts with a large number of receptors on the surface of macrophages in tumor microenvironment, potentially regulating the role of macrophages to facilitate the growth of tumor cells. As an important angiogenic factor, haptoglobin can stimulate the growth of blood vessels. It can promote the growth and differentiation of endothelial cells and the development of collateral vessels of neovascularization, and provide blood supply for cancer lesions, thereby promoting the growth of tumor cells and tumor enlargement 37.

By mass spectrometry and ELISA analyses, we found that the serum level of haptoglobin in prostate cancer with bone metastasis and non-metastasis groups were significantly different. However, immunohistochemical analysis of prostate cancer tissues indicated that there was no significant difference in the mean optical density between the two groups. The reason for this inconsistency could be because haptoglobin is mainly synthesized by the liver, and in addition to its secretion by tumor cells, other factors regulating its synthesis in the liver may regulate the content of haptoglobin in the blood. Therefore, the regulation mechanism of the final concentration of haptoglobin in blood is very complicated. Although immunohistochemical analysis showed that the expression of haptoglobin in bone metastasis of prostate cancer was significantly higher than that in prostate cancer tissues, analysis of its expression in three cell lines (DU145, LNCaP and PC3) derived from different metastasis of prostate cancer indicated that PC3, which is derived from bone metastasis, had the lowest expression. This indicates that the expression of haptoglobin in prostate cancer cells is influenced by the microenvironment in different metastatic sites. The specific mechanism needs further study.

Tetranectin, encoded by the clec3b gene and belonging to the C type lectin family, is a plasminogen kringle-4 binding protein that can be detected in the plasma and the extracellular matrix. In plasma or serum of healthy adults, the mean concentration of tetranectin is 10.8 mg/L (9-12.1 mg/L) 38. Studies have shown that tetranectin is expressed in a variety of neuroendocrine tissues, epithelial and mesenchymal cells, such as fibroblasts, monocytes and neutrophils. Tetranectin has been closely related to a variety of diseases since it was first isolated and purified from human plasma in 1986 39-43. Although studies on tetranectin have been carried out for more than 30 years, there are only few reports on tetranectin and its biological functions with the underlying mechanisms remain unclear. Some studies suggest that tetranectin may play an important role in tissue remodeling and mineralization of bone during osteogenesis 44. Indeed, tetranectin gene knockout mice display delayed union of fracture and wound healing 45, 46. Some research reports that plasma tetranectin levels are reduced in malignant tumors and the plasma tetranectin concentration decreases in parallel with the growth of some tumours 43, 47, 48. Consistent with this trend, our study found that the serum concentrations of tetranectin was found progressively lower going from prostate hyperplasia, prostate cancer without metastasis and prostate cancer with bone metastasis groups. In DU145, LNCaP and PC3, the expression of tetranectin was lowest in the PC3 cells obtained from bone metastasis, suggesting that tetranectin has a high correlation with bone metastasis of prostate cancer.

Chen et al. reported that high intratumoral expression of tetranectin is associated with poor prognosis of patients with gastric cancer after gastrectomy 49. The alteration of tetranectin expression in cancer tissue is also considered as a predictive biomarker for prognosis in breast, bladder, oral and ovarian cancer 43, 48, 50, 51. However, previous studies have failed to distinguish differences in tetranectin expression between epithelial and mesenchymal cells. In our immunohistochemical analysis, we found that the expression of tetranectin in the epithelial cells of bone metastasis tissues, prostate cancer tissues with bone metastasis, prostate cancer tissues without metastasis and prostatic hyperplasia tissues decreased in that order. But in the mesenchyme, the expression was found progressively increased going from prostate cancer tissues with bone metastasis, prostate cancer tissues without metastasis, and prostate hyperplasia tissues. It has been reported that tetranectin plays an important role in regulating the fibrinolysis and proteolytic procedures by binding to kringle-4 of circulating plasminogen to enhance activation of plasminogen into plasmin 46. Our hypothesis is that with an increase in malignancy, the process of tissue remodeling and angiogenesis consumes more tetranectin, leading to the decrease of tetranectin in the mesenchyme and blood. Simultaneously, there is an increase of tetranectin expression in prostate cancer cells to promote the growth and metastasis of tumor. Further research is needed to confirm this and the underlying mechanisms of tetranectin function in human malignancies.

## 5. Conclusion

Bone metastasis of prostate cancer is a complex biological process, involving a variety of gene and protein changes. In this study, 32 DEPs related to bone metastasis of prostate cancer were identified by proteomic analysis of serum samples from prostate cancer patients. Three such DEPs with significant fold changes were validated in prostate cancer tissues and serum samples, respectively. Moreover, we analyzed the expression of the three proteins in the three commonly used prostate cancer cell lines to provide a basis for the selection of cell lines for further in vitro study of bone metastasis mechanism. However, the proteins associated with bone metastasis of prostate cancer may not be all detectable in serum, and local pathological and physiological changes in prostate tissue may be important. Meanwhile, a large proportion of the identified DEPs could be secondary changes. Therefore, further screening is essential to identify key proteins and signal pathways, in order to design novel diagnostic and therapeutic approaches for the treatment of prostate cancer with bone metastasis.

### Funding

This work was mainly supported by the joint fund of Guizhou provincial Science & Technology Department and Guizhou provincial People's Hospital [LH(2016) 7164], and partially supported by the joint fund of Guizhou provincial Science & Technology Department and the Affiliated Hospital of Guizhou Medical University [LH(2015) 7392].

## Figures and Tables

**Table 1:** Information of differentially expressed proteins identified by LC-MS/MS

Accession No	Gene Symbol	Protein Name	C.V	Ratio	P Value	Flag
P06396	GSN	Gelsolin	0.30	0.51	0.011	Down
P07996	THBS1	Thrombospondin-1	0.20	0.63	0.015	Down
P29622	SERPINA4	Kallistatin	0.19	0.51	0.003	Down
P10643	C7	Complement component C7	0.20	0.72	0.042	Down
P04196	HRG	Histidine-rich glycoprotein	0.46	0.51	0.036	Down
P02750	LRG1	Leucine-rich alpha-2-glycoprotein	0.13	2.41	0.005	Up
P11226	MBL2	Mannose-binding protein C	0.01	0.60	0.000	Down
P05452	CLEC3B	Tetranectin	0.32	**0.37**	0.003	Down
P55058	PLTP	Phospholipid transfer protein	0.18	0.75	0.046	Down
Q96IY4	CPB2	Carboxypeptidase B2	0.05	0.61	0.000	Down
P22352	GPX3	Glutathione peroxidase 3	0.02	0.61	0.000	Down
P05160	F13B	Coagulation factor XIII B chain	0.22	0.65	0.023	Down
P04278	SHBG	Sex hormone-binding globulin	0.09	0.59	0.001	Down
P02747	C1QC	Complement C1q subcomponent subunit C	0.02	0.72	0.000	Down
P02741	CRP	C-reactive protein	0.09	3.82	0.001	Up
P0DJI9	SAA2	Serum amyloid A-2 protein	0.28	3.10	0.026	Up
P20742	PZP	Pregnancy zone protein	0.17	2.27	0.010	Up
P13647	KRT5	Keratin, type II cytoskeletal 5	0.21	0.67	0.025	Down
Q15166	PON3	Serum paraoxonase/lactonase 3	0.12	0.67	0.005	Down
P12259	F5	Coagulation factor V	0.16	0.69	0.017	Down
P00738	HP	Haptoglobin	0.61	**7.69**	0.088	Up
O75882	ATRN	Attractin	0.08	0.66	0.001	Down
O43866	CD5L	CD5 antigen-like	0.14	1.44	0.035	Up
P13987	CD59	CD59 glycoprotein	0.22	**6.33**	0.007	Up
Q15582	TGFBI	Transforming growth factor-beta induced protein	0.14	0.68	0.010	Down
P60709	ACTB	Actin, cytoplasmic 1	0.03	0.70	0.000	Down
P18428	LBP	Lipopolysaccharide-binding protein	0.06	1.43	0.004	Up
Q9NPH3	IL1RAP	Interleukin-1 receptor accessory protein	0.31	0.58	0.026	Down
P15814	IGLL1	Immunoglobulin lambda-like polypeptide 1	0.12	1.53	0.015	Up
P49908	SEPP1	Selenoprotein P	0.08	0.76	0.007	Down
P62805	HIST1H4A	Histone H4	0.15	1.71	0.017	Up
Q02153	GUCY1B3	Guanylate cyclase soluble subunit beta-1	0.08	1.84	0.002	Up
